# AbobotulinumtoxinA (Dysport^®^), OnabotulinumtoxinA (Botox^®^), and IncobotulinumtoxinA (Xeomin^®^) Neurotoxin Content and Potential Implications for Duration of Response in Patients

**DOI:** 10.3390/toxins10120535

**Published:** 2018-12-13

**Authors:** Malgorzata Field, Andrew Splevins, Philippe Picaut, Marcel van der Schans, Jan Langenberg, Daan Noort, Keith Foster

**Affiliations:** 1 Ipsen Bioinnovation, Abingdon OX14 4RY, UK; gosia.olszowka@gmail.com (M.F.); andrew.splevins@ipsen.com (A.S.); 2 Ipsen Pharma, Cambridge, MA 02142, USA; philippe.picaut@ipsen.com; 3 TNO—CBRN Protection, 2288GJ Rijswijk, The Netherlands; marcel.vanderschans@tno.nl (M.v.d.S.); jan.langenberg@tno.nl (J.L.); daan.noort@tno.nl (D.N.)

**Keywords:** botulinum toxin, BoNT, spasticity, Dysport^®^, abobotulinumtoxinA, glabellar lines

## Abstract

Botulinum neurotoxin type-A (BoNT-A) blocks the release of acetylcholine from peripheral cholinergic nerve terminals and is an important option for the treatment of disorders characterised by excessive cholinergic neuronal activity. Several BoNT-A products are currently marketed, each with unique manufacturing processes, excipients, formulation, and non-interchangeable potency units. Nevertheless, the effects of all the products are mediated by the 150 kDa BoNT-A neurotoxin. We assessed the quantity and light chain (LC) activity of BoNT-A in three commercial BoNT-A products (Dysport^®^; Botox^®^; Xeomin^®^). We quantified 150 kDa BoNT-A by sandwich ELISA and assessed LC activity by EndoPep assay. In both assays, we assessed the results for the commercial products against recombinant 150 kDa BoNT-A. The mean 150 kDa BoNT-A content per vial measured by ELISA was 2.69 ng/500 U vial Dysport^®^, 0.90 ng/100 U vial Botox^®^, and 0.40 ng/100 U vial Xeomin^®^. To present clinically relevant results, we calculated the 150 kDa BoNT-A/US Food and Drug Administration (FDA)-approved dose in adult upper limb spasticity: 5.38 ng Dysport^®^ (1000 U; 2 × 500 U vials), 3.60 ng Botox^®^ (400 U; 4 × 100 U vials), and 1.61 ng Xeomin^®^ (400 U; 4 × 100 U vials). EndoPep assay showed similar LC activity among BoNT-A products. Thus, greater amounts of active neurotoxin are injected with Dysport^®^, at FDA-approved doses, than with other products. This fact might explain the long duration of action reported across multiple indications, which benefits patients, caregivers, clinicians, and healthcare systems.

## 1. Introduction

Botulinum neurotoxins (BoNTs) are a well-established treatment option for disorders such as dystonia or spasticity [1], as well as for aesthetic facial treatments [2]. BoNTs cause temporary muscle relaxation, which can ease symptoms and aid rehabilitation [3] when injected into specific muscles of patients suffering from movement disorders. This action is a result of their potent ability to inhibit neurotransmitter release, which causes flaccid paralysis [4,5,6]. This action underpins the therapeutic and aesthetic use of the toxin. [5,6]. BoNT also inhibits acetylcholine release at autonomic nerve terminals and is used in hyperhidrotic and urological disorders [7].

There are multiple serotypes of BoNTs. Currently, all but one of the therapeutic BoNT products are botulinum neurotoxin type-A (BoNT-A). At present, there are three BoNT-A products available worldwide: abobotulinumtoxinA (Dysport^®^, Ipsen, Paris, France), onabotulinumtoxinA (Botox^®^, Allergan, Irvine, CA, USA), and incobotulinumtoxinA (Xeomin^®^, Merz Pharmaceuticals GmbH, Frankfurt, Germany) [4]. There are also other BoNT products, but they are currently only available in limited markets or territories around the world [8]. In this study, we have only compared the three BoNT-A products that are available worldwide. Each of these BoNT-A products has a unique manufacturing process and contains different excipients [8]. 

Despite differences among these three products, the therapeutic effects are mediated in all instances by the 150 kDa BoNT-A neurotoxin, which consists of a light chain (LC) and a heavy chain (HC). The HC drives neuron-specific binding (H_C_ domain) and translocation (H_N_ domain) of the toxin LC into the neuronal cytosol. The LC contains the proteolytic domain and is responsible for the catalytic activity and substrate specificity of the toxin within the neuron [6,9]. 

Due to the differences in BoNT-A products, each has its own dosing guidelines based on potency units, originally defined through murine lethality tests [10]. The lethality tests underpinning unit definitions differ for the various products and are sensitive to various factors, including the mouse strain used, their age and sex, the animal housing conditions (such as light/dark cycles and feeding times), experimental injection time, and diluent buffer [11,12]. This variability has resulted in potency units that are specific to each product and are not interchangeable [11,13,14,15]. Due to this non-equivalence, the recommended number of units varies greatly between products. For example, in adult upper limb spasticity (AUL), the total recommended doses according to US Food and Drug Administration (FDA) labels are as follows: Dysport^®^ 1000 U, Botox^®^ 400 U, and Xeomin^®^ 400 U; for the aesthetic glabellar lines (GL) indication, approved doses are Dysport^®^ 50 U, Botox^®^ Cosmetic 20 U, and Xeomin^®^ 20 U [13,14,15].

The effectiveness of BoNT-A treatment in both therapeutic and aesthetic indications is the result of many factors, including the extent of disease severity or patient disability and treatment objectives, as well as injection technique (including dose and number of injected muscles), injector experience, and the holistic treatment approach, ensuring sufficient rehabilitation and support alongside BoNT-A injections [16,17,18,19,20]. At the molecular level, there are other factors that may affect treatment effectiveness. The ability of the BoNT-A to bind, internalise, and deliver the LC into the target neuron plays a role, as does the proteolytic activity of the LC (the rate at which it cleaves its SNARE (Soluble NSF Attachment Protein) REceptor) protein target and thus blocks neurotransmitter release). The quantity of BoNT-A available also significantly impacts therapeutic effectiveness: the greater the number of 150 kDa BoNT-A molecules, the greater the ability to cleave SNARE substrates [21]. The total Clostridial protein content of the three main commercially available BoNT-A products—Dysport^®^, Botox^®^, and Xeomin^®^—has been previously reported in the literature, giving values of 5 ng per 100 U vial of Botox, 4.35 ng per 500 U vial of Dysport (correcting an earlier publication stating 12.5 ng per 500 U vial), and Xeomin containing 0.6 ng per 100 U vial [13,22,23]. As noted by Frevert [24], however, these values represent not only the core neurotoxin but also the complexing proteins when present. With regard to neurotoxin activity and, therefore, clinical activity, it is the core 150 kDa BoNT-A content that is important. The 150 kDa BoNT-A content in Dysport, Botox, and Xeomin was first measured and reported by Frevert [24] using a sensitive sandwich ELISA. Our research extends the work of Frevert by comparing both BoNT-A quantity and proteolytic activity among the three main commercially available BoNT-A products. Other aspects of neurotoxin function are not assessed in this study. 

The differences in potency units mean that the quantity of neurotoxin in each product cannot be directly compared, since one Dysport^®^ unit does not equal one Botox^®^ unit, which does not equal one Xeomin^®^ unit. Each BoNT-A product does, however, contain the 150 kDa neurotoxin, with different products containing different amounts. To make a direct comparison of the amount of neurotoxin (150 kDa BoNT-A) in each product, we assayed the quantity of 150 kDa neurotoxin protein (in nanograms) in each vial of Dysport^®^, Botox^®^, and Xeomin^®^ using a sandwich enzyme-linked immunosorbent assay (ELISA) with BoLISA^®^ antibodies, and compared this with the labelled unit strength. 

To investigate the LC activity of neurotoxin within each BoNT-A product, we used the EndoPep method in which a specific substrate peptide is cleaved by the toxin [25]. The concentration of cleaved peptide (product peptide) after a certain incubation time is measured and the concentration of product peptide obtained with the BoNT-A products versus different concentrations of reference BoNT-A is compared [26]. The quantity of reference BoNT-A giving an equivalent level of cleavage to that by a product BoNT-A is then taken as a measure for the activity of the LC in the product BoNT-A. In the original paper from Kalb et al. [26], BoNTs were isolated from food matrixes using antibody-loaded magnetic beads, followed by the EndoPep reaction. In our study, we decided to skip the isolation step for a number of reasons:

1. We anticipated that the concentration of BoNT-A in the samples would be high enough that a sample enrichment would not be necessary.
2. The matrix of the samples is relatively clean, making an extra clean-up step redundant.
3. The isolation of BoNT-A with antibodies introduces another possible source for variation, as it is unknown whether the affinity of the antibody for BoNT-A in the different samples is affected by the different compositions of the product formulations, and an additional sample manipulation.

Therefore, the solutions of the toxin products were mixed directly in the EndoPep mixture. 

We also decided to assay the cleaved peptides with capillary electrophoresis and laser-induced fluorescence (CE-LIF) detection, using fluorescently labelled substrate peptides, rather than by using a mass spectrometric read-out. We use this method routinely in our laboratory (see Van Uhm et al., [27]) because of its robustness, ease of operation, and easily quantifiable spectrometric read-out. 

Our results revealed greater amounts of BoNT-A neurotoxin with Dysport^®^, compared with Botox^®^ and Xeomin^®^, when used at the FDA-recommended doses for the treatment of AUL, adult lower limb spasticity (ALL), and GL. Since all three BoNT-A products are approved for use in these indications [13,14,15], we were able to do a direct comparison of approved doses. Furthermore, the EndoPep assay showed no significant differences in LC activity between the BoNT-A present in Dysport^®^, Botox^®^, and Xeomin^®^. Therefore, when Dysport^®^ is used at the recommended doses, more active neurotoxin is administered than when using Botox^®^ or Xeomin^®^. Given that the duration of BoNT-A muscle paralysis is known to be dependent on the quantity of active neurotoxin applied [28], this greater amount of active BoNT-A in Dysport^®^ at the recommended dose potentially prolongs post-injection denervation and duration of action, relative to other products.

## 2. Results

### 2.1. Quantity of 150 kDa BoNT-A

We analysed three batches of Dysport^®^, Botox^®^, and Xeomin^®^ by sandwich ELISA on three separate occasions using a pair of antibodies specific to the 150 kDa BoNT-A protein. We measured the quantity of BoNT-A by extrapolation against a standard curve of recombinant BoNT-A (rBoNT-A) analysed in the same assay. We have previously demonstrated that the activity of this rBoNT-A is identical to that of purified natural BoNT-A obtained from commercial sources [29,30].

Table 1 shows the mean quantity of 150 kDa neurotoxin per vial measured for each product and each batch, tested over three runs. The average amount of 150 kDa neurotoxin for each product is also shown. In a 500 U Dysport^®^ vial, there was 2.69 ± 0.03 ng of BoNT-A; in addition, there were 0.90 ± 0.03 ng of BoNT-A in a 100 U vial of Botox^®^ and 0.40 ± 0.01 ng in a 100 U vial of Xeomin^®^.

We used the mean ng/vial values to calculate the amount of toxin (pg) per potency unit. The mean pg per unit value (±SD) was 5.38 ± 0.07 for Dysport^®^, 9.04 ± 0.3 for Botox^®^, and 4.03 ± 0.06 for Xeomin^®^ (Table 2).

To present results in a clinically relevant manner, we calculated the amount of BoNT-A in the maximum recommended dose for AUL and ALL spasticity and for GL. For AUL, this equated to 5.38 ng Dysport^®^ (1000 U dose; 2 × 500 U vials), 3.62 ng Botox^®^ (400 U dose; 4 × 100 U vials), and 1.61 ng Xeomin^®^ (400 U dose: 4 × 100 U vials). In GL, this equated to 0.27 ng Dysport^®^ (50 U dose), 0.18 ng Botox^®^ (20 U dose), and 0.08 ng Xeomin^®^ (20 U dose). For these and other calculated quantities, please refer to Table 3. 

### 2.2. Light Chain Activity of BoNT-A Products

We analysed three vials from one batch each of Dysport^®^ (batch L23919, expiry date December 2018), Botox^®^ (batch C4289C3, expiry date September 2019), and Xeomin^®^ (batch 694458, expiry date July 2019) to assess LC activity using the EndoPep method, in which each BoNT-A product is incubated with a fluorescent-labelled substrate peptide. On this occasion, we chose to use Dysport^®^ vials from a 300 U batch so that the amount of toxin in the vial was more similar to those in the Botox and Xeomin vials, as established in the quantitation work. After 4 h of incubation, we measured LC activity by looking at the concentration of cleaved target peptide by CE-LIF detection. We quantified LC activity against a standard curve of a rBoNT-A protein control, allowing the derivation of the quantity of recombinant neurotoxin (in ng) required to achieve equivalent LC activity to one vial of each product. 

We also analysed the same batches of each BoNT-A product by sandwich ELISA using a pair of antibodies specific to the 150 kDa BoNT-A protein and the quantity of BoNT-A measured by extrapolation against a standard curve of rBoNT-A analysed in the same assay, as detailed above. The results are shown in Table 4.

The resulting ratio of activity quantity (the relative quantity of rBoNT-A giving the same protease activity assessed by the EndoPep method) to protein quantity (ELISA) was 0.79 (±0.17) for Dysport^®^, 1.08 (±0.23) for Botox^®^, and 0.79 (±0.05) for Xeomin^®^ (given as the mean (±SD)). We assessed these ratios statistically for any differences between the products in case of differing specific LC activities.

Table 5 summarises the relative LC activity per nanogram of neurotoxin among the BoNT-A products. Mean differences in activity vary only slightly, from 0 ± 0.18 to 0.29 ± 0.29, and all differences were non-significant. Thus, these results demonstrate that there are no significant differences in LC activity among the BoNT-A products, and the 150 kDa neurotoxin molecules in each product are equally active. 

## 3. Discussion

Our analyses reveal that there are notable differences in the quantity of neurotoxin in each potency unit of different commercial BoNT-A products (Dysport^®^, Botox^®^, and Xeomin^®^), which, in turn, results in large differences in the amount of BoNT-A 150 kDa protein in a vial of each product. This work confirms the differences and non-interchangeability of BoNT-A product potency units.

We determined the LC activity of BoNT-A products by the EndoPep method, using CE-LIF for analysis of the cleaved substrate peptides [27]. CE-LIF proved to be sufficiently sensitive, and we were able to inject the EndoPep mixture without any further sample preparation. By measuring the amount of the peptide cleaved by each product, we were able to compare the relative LC activities across products. The original paper from Kalb et al. [26] used a mass spectrometric analysis, namely MALDI-TOF. MALDI-TOF has some attractive aspects, such as a fast analysis and a high specificity. In our hands, however, MALDI-TOF was not sensitive enough for these samples using the substrate peptide described in Kalb et al. [26]. Recently, Wang et al. [32] published a paper with peptide substrate sequences that improve the sensitivity of the detection of BoNT/A. At the time that the Wang et al. paper appeared, however, we were already conducting studies with the CE-LIF method, and the substrate used provided the necessary sensitivity for this purpose. In future studies it may be worth assessing the further optimised substrate peptides mentioned in [32] with MALDI-TOF detection as well. Alternatively, the samples could be analysed with liquid chromatography–mass spectrometry. The large content of bovine serum albumin (BSA) in the EndoPep assay sample and longer time of analysis, however, made this option less suitable. To make a true comparison among the various BoNT-A products (which are each formulated in differing excipients), we designed the incubation and sampling scheme in such a way that all products were in an identical incubation mixture during the EndoPep assay. In this way, the relative enhancing or inhibitory effect of a buffer constituent or other excipient is equally possible for all products tested. 

The FDA approves doses of BoNT-A products based on the doses that have been shown to be safe and effective in clinical trials. At the FDA-approved doses, the data in Table 3 show that a greater amount of neurotoxin is injected with Dysport^®^ than with other BoNT-A products. For example, the amount of BoNT-A per FDA maximum approved dose for AUL spasticity equates to 5.38 ng Dysport^®^ (in 1000 U: 2 × 500 U vials), 3.62 ng Botox^®^ (in 400 U: 4 × 100 U vials), or 1.61 ng Xeomin^®^ (in 400 U: 4 × 100 U vials). We also observed similar differences at the approved doses in the aesthetic indication of GL. Despite these differences in BoNT-A, when used at the FDA-recommended doses, all three BoNT-A products have a similar safety profile [13,14,15]. We were not able to compare all products for adult lower limb spasticity, since Xeomin^®^ is not currently approved in the U.S. for this indication, following results from a recent Phase 3 study (NCT01464307) [31]. 

We measured the quantity of BoNT-A in both 300 and 500 U vials of Dysport^®^ and found that the values obtained were consistent: 5.38 (±0.07) pg/U for the 500 U vial and 6.02 (±0.39) pg/U for the 300 U vial. Thus, conclusions regarding the quantity of toxin injected relative to other products based on values obtained for the 500 U vial are also applicable to the 300 U vial.

Our analyses also revealed that the 150 kDa neurotoxin molecules within the different BoNT-A products had equivalent LC activities. Thus, one molecule of Dysport^®^ had the same LC activity as one molecule of Botox^®^ or Xeomin^®^. This is perhaps unsurprising given that all products were produced by a Hall strain of *Clostridium botulinum* bacteria and contain the A1 subtype of BoNT-A. Given these results, we can discount differences in LC activity as being responsible for observed differences between the BoNT-A products. Potential effects of the different formulations upon the proteolytic activity measured in this study, which we addressed by standardising the composition of the assay buffer among the BoNT-A samples measured, are unlikely to be of relevance to in vivo activity in the therapeutic context due to the dilution and diffusion of the formulation components likely to occur upon reconstitution and injection into the tissue. Although we did not measure other aspects of BoNT-A activity, we believe that our results are consistent with there not being significant differences in BoNT-A activity between the products. In this regard, our data build on those published by Frevert in 2010 [24]. The amount of neurotoxin per vial shown in our analysis confirms the data published by Frevert. Our experiments, however, extend this analysis by also investigating the LC activity of neurotoxin in the various products. The conclusions in the Frevert paper are not supported by the data obtained in this study, where no differences in specific LC activity were observed between the products. Frevert, in contrast, proposed that based upon the unit activity of the products, Xeomin^®^ had a greater specific activity than the other products. Given the uniqueness of potency units to each BoNT-A product, however, specific activities cannot be calculated and compared among products based upon the unit activities of the different products. A standardised assay of the full toxin functionality per mass unit is required for this assessment. Our results indicate that the LC activity of the BoNT-A in each of the three main products does not significantly differ; in other words, each product has equivalent LC activity per quantity of BoNT-A.

During clinical trials of Dysport^®^ in adults with upper limb or lower limb spasticity and in children with lower limb spasticity, repeated injections of Dysport^®^ resulted in improvements in active movement and function [33,34,35,36,37]. These improvements are rarely seen in the BoNT literature. The safety profile demonstrated in these studies was similar to those observed with other BoNT-A products when dosed as per their labels. It is important, therefore, that our comparisons of levels of BoNT-A injected in various clinical indications between the different products is performed in the context of the recommended dose. Similarly, the context of the recommended dose also ensures that there is no increased immunogenicity risk based on the quantities of BoNT-A being administered, as it has been shown that the incidence of neutralising antibody formation is low for all of the products when dosed as per their label [38]. 

The current BoNT-A prescribing information states that administration should not be performed at intervals of less than 12 weeks [13,14,15,39], but few studies have carefully assessed the time to retreatment following repeated injections of BoNT-A. Both of the recent trials in patients with adult spasticity mentioned above had flexible reinjection visits, and many patients treated with Dysport^®^ did not require reinjection until much later than the standard 12 weeks (36.9% of patients in the open-label adult upper limb spasticity trial; 20.1% of patients in the open-label adult lower limb spasticity trial) [33,34,35], a result also observed in the recent trial of Dysport^®^ in children with lower limb spasticity (74.0% of patients in the double-blind, paediatric, lower limb spasticity trial) [36]. It is well known that the amount of active BoNT-A injected is positively correlated to the duration of action (muscle paralysis) of the treatment. This dose–duration relationship has been clearly demonstrated in animal models [28], and a dose–efficacy relationship has been seen in patients with upper limb spasticity, though duration of response was not explored in this study [40]. Other clinical studies from the field of aesthetic medicine, however, have provided evidence for a dose–duration relationship [41,42,43]. Given this relationship, and the results shown here, we hypothesise that the greater number of 150 kDa neurotoxin molecules of BoNT-A in the doses of Dysport^®^ injected during the aforementioned studies allowed a prolonged duration of action. In turn, this led to a requirement for less frequent injections. Importantly, clinical improvements and a prolonged duration of action were observed at well-tolerated doses in these studies. 

These findings may have important consequences for patients who are currently treated with BoNT-A, since a long duration of response with Dysport^®^ has many potential benefits. Importantly, with a long duration of response, patients could receive a longer period of sustained symptom relief between injections, rather than experiencing a waning of clinical effect and reoccurrence of symptoms in the weeks preceding a repeat injection. In some cases, a long duration of response may enable the injection of additional muscles at Week 12, when the original muscles are still benefiting from the previous injection. Finally, patients may require less frequent and fewer injections, resulting in less disruption to work and social lives. The reduced cost burden on healthcare systems is also worth noting.

## 4. Conclusions

There are notably greater amounts of active neurotoxin in Dysport^®^ when used at the total FDA-recommended dose for AUL, ALL, and GL than in Botox^®^ and Xeomin^®^. This greater amount of active neurotoxin may prolong the block of neurotransmitter release at the neuromuscular junction following Dysport^®^ injection and may result in a clinically longer duration of action that benefits patients, caregivers, and healthcare systems.

## 5. Materials and Methods 

### 5.1. General Methods

Capture and detection antibodies were purchased from BioSentinel, Inc. (BoLISA^®^ #A1029) (Madison, WI, USA). Plates were washed three times between incubations using a buffered solution made from tablets comprising 0.01 M phosphate, 0.0027 M KCl, 0.137 M sodium chloride (NaCl), pH 7.4, and 0.05% phosphate-buffered saline with Tween^®^-20 (PBS-T). Recombinant BoNT-A1 was expressed and purified in-house. DNA encoding rBoNT-A1 (NCBI Reference Sequence accession number: WP_011948511.1; UniParc identifier: UPI0000001386) was synthesised and optimised for expression in *Escherichia coli*. This was then transformed into BLR (DE3) *Escherichia coli* cells (Novagen Cat# 69053-3, Lot D00088845) (Novagen, Madison, WI, USA) cultured in 1 litre batches. Purification of rBoNT-A1 (SXN102342) was achieved by selective precipitation, followed by hydrophobic interaction and ion exchange column chromatography, coupled with an endoproteinase activation step. The activation stage cleaves the expressed single-chain, precursor protein at a specific recognition site and produces the fully active di-chain rBoNT-A1 product, the light and heavy chains being joined by a disulphide bond. Hydrophobic interaction chromatography was used to separate any residual endoproteinase from the target product.

### 5.2. BoLISA^®^ Procedure

The mass quantity (ng) of 150 kDa BoNT-A in commercial products (Dysport^®^, Botox^®^, and Xeomin^®^) was determined using a sensitive sandwich ELISA (BoLISA^®^) using a pair of mouse monoclonal anti-BoNT-A antibodies, one of them biotinylated. 

A microwell plate (Thermo Scientific Nunc^®^ #439454) (Abingdon, Oxon, UK) was coated with BoLISA^®^ capture antibody diluted to 2 µg/mL in phosphate-buffered saline (PBS, Gibco™ #10010023) (Thermo Fisher, Abingdon, Oxon, UK) and incubated for 1 h at 37 °C. After incubation, the plate was washed three times with PBS-T and incubated with blocking reagent (Blocker™ Casein in PBS, Thermo Scientific #37528) for 1 h at 37 °C. During the incubation time, samples and standards were prepared. Samples were prepared by reconstitution of the lyophilised products in the vial. Vials of Dysport^®^ (500 U and 300 U) and Botox^®^ (100 U) were reconstituted with 1 mL of blocking reagent and Xeomin^®^ (100 U) with 0.5 mL. Xeomin was reconstituted in a smaller volume to ensure there was sufficient concentration of the product to be in the quantitation range for the assay. Standards were prepared by serial dilution of the stock rBoNT-A (2.4 mg/mL) with the blocking reagent. Intra-assay quality controls were prepared and run alongside the test samples to assess the accuracy of the assay. Test samples, standards, and quality control samples were pipetted in triplicate onto the assay plate and incubated for 1 h at 37 °C. Wells were washed a further three times with PBS-T, and the plate was incubated with the biotinylated detection BoLISA^®^ antibody (BioSentinel Inc., Madison, WI, USA) and diluted to 2 µg/mL in blocking reagent for 1 h at 37 °C. Excess detection antibody was removed using another set of three washes, and horseradish peroxidase (HRP)-conjugated streptavidin (Pierce™ Streptavidin Poly-HRP, Thermo Scientific #21140) at 0.2 µg/mL, diluted in blocking reagent, was added onto the plate. After the final wash step, colour reactions were developed using 3,3′,5,5′-tetramethylbenzidine (TMB) One Component HRP Microwell Substrate (BioFX Laboratories #TMBW-1000-01) (Owings Mills, MA, USA) and subsequently stopped after 2 minutes with the addition of 450 nm Stop Reagent for TMB (BioFX Laboratories #STPR-1000-01). The absorbance of each well was read at 450 nm using a microwell plate reader (Synergy HT, BioTek, Winooski, VT, USA). The amount of the 150 kDa neurotoxin in the three commercial products was determined from the mean of three independent assays. 

### 5.3. Standard Curve of the Sandwich ELISA

**Figure 1:**
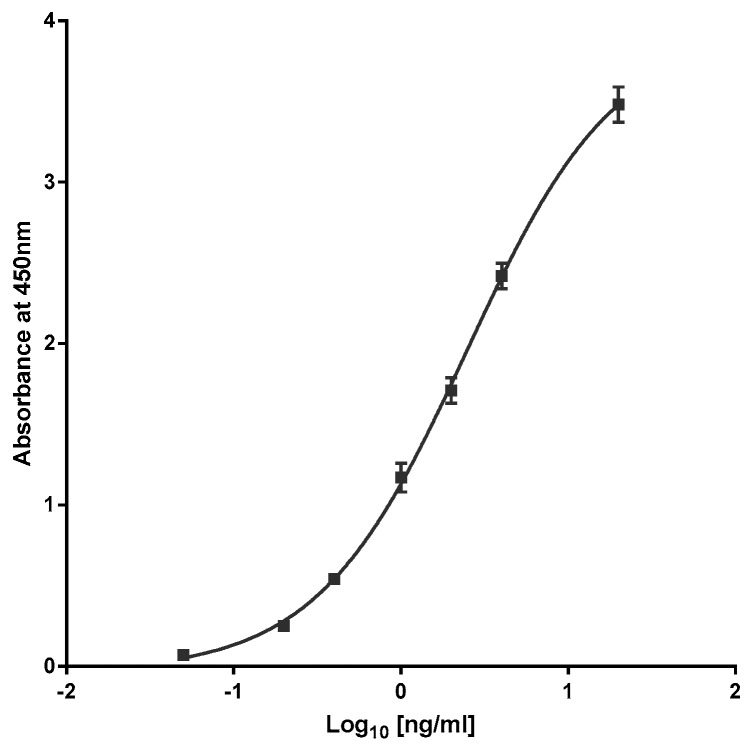
Representative standard curve obtained using BoLISA^®^ antibody pair detection of recombinant BoNT-A1.

Recombinant BoNT-A1 was used to generate a standard curve from 20 to 0.05 ng/mL. Data were analysed using GraphPad Prism software (version 7.04, GraphPad Software, Inc, San Diego, CA, USA) and fitted to a Sigmoidal 4PL curve. A representative standard curve is presented in Figure 1. The quantities of 150 kDa BoNT-A1 in each sample were determined by interpolation of the sample absorbance at 450 nm against the standard curve in each assay. The means and SDs of three results were calculated from these determined values (Table 1 and Table 2). 

### 5.4. ELISA Accuracy and Specificity

An assay accuracy of ±30% was obtained via the intra-assay quality controls in the range of 0.5‒2 ng/mL concentration, providing a suitable tool to test the commercial products. Interference by excipients was tested and confirmed that the formulations present in the commercial products had no impact on the accurate detection of BoNT-A1. Products were resuspended in Blocker™ Casein in PBS pH 7.4 to ensure the rapid dissociation of complexing proteins [44]. 

### 5.5. EndoPep Assay

#### 5.5.1. Chemicals

HEPES (4-[2-Hydroxyethyl]-1-piperazine ethanesulfonic acid, zinc chloride, sodium hydroxide, DTT (1,4-Dithio-DL-threitol), boric acid, bovine serum albumin (BSA), citric acid, lactose, sucrose, NaCl, and fluorescein were purchased from Sigma-Aldrich (Bornem, Belgium). Substrate peptide, the fluorescent-labelled substrate peptide 6FAM-RGSNKPKIDAGNQRATRXLGGR-NH2, and the fluorescent-labelled product peptide 6FAM-RGSNKPKIDAGNQ were purchased from PepScan (Lelystad, The Netherlands).

#### 5.5.2. EndoPep Procedure

A volume of 200 µL of product-specific reconstitution buffer was added to the vial of the BoNT-A product by injecting the liquid through the septum of the vial. The vial was weighed before and after addition of the buffer to determine the exact volume. The different products contain different excipients at different concentration. In order to avoid possible enhancement or inhibitory effects, the composition of the reconstitution buffer was different for each product in order to produce the same final reaction conditions for all samples. Table 6 shows the excipients for each product and the corresponding composition of each product-specific reconstitution solution. The reconstituted products were therefore all in a final buffer composition identical to that given for the r-BoNT-A reconstitution buffer in Table 6.

The reconstituted botulinum toxin sample (5 μl) was mixed with 80 μl EndoPep reaction buffer (20 mM HEPES pH 7.3, 200 μM ZnCl2, 1 mg/mL BSA, and 10 mM DTT). Fluorescent-labelled substrate peptide 6FAM-RGSNKPKIDAGNQRATRXLGGR-NH2 (40 μl, 200 μM dissolved in reaction buffer) was added, and the mixture was incubated in a thermal mixer at 36 °C for 4 h. The end volume was always 125 µL. After 4 h, 10 µL of the internal standard solution (Fluorescein at 4.68 µM in water) was added, and the mixture was cooled in water-ice in order to stop the cleavage reaction. No more than six samples were processed at a time so that they could all be measured within 90 minutes with CE-LIF.

#### 5.5.3. Capillary Electrophoresis

Capillary electrophoresis experiments were performed on a P/ACE MDQ capillary electrophoresis instrument equipped with LIF detection (Beckman Instruments, Fullerton, CA, USA). The excitation wavelength was 488 nm, and the emission wavelength was 520 nm. The total length of the fused silica capillary (i.d. 75 μm, PolyMicro, Phoenix, AZ, USA) was 70 cm; the effective length to the detector was 60 cm. The separation voltage was 25 kV. Samples were introduced by pressure injection for 5 seconds at 0.5 psi. The running buffer consisted of 100 mM sodium borate buffer at pH 8.9. 

### 5.6. Statistical Assessment of the Ratio of BoNT-A Quantity Obtained by ELISA and EndoPep Assays

For each product, the ratio was calculated. The variance of the ratio was estimated by the Delta method. To compare the ratio with another ratio, the difference and variance were calculated, then the Z score was found, and, finally, the probability that the difference is equal to zero was determined. After that, a Sidak adjustment was performed to take into account the multiplicity of tests. Differences were considered significant when the *p* value was <0.05.

## Figures and Tables

**Table 1:** Quantity (ng) of 150 kDa BoNT-A in Dysport^®^, Botox^®^, and Xeomin^®^, analysed by ELISA using BoLISA^®^ antibodies.

Product	Batch	Expiry Date	Quantity of BoNT-A (ng/vial)	
			Batch, Mean (SD)	Product, Mean (SD)
Dysport^®^ 500 U	M00405	Dec. 2018	2.73 (0.20)	2.69 (0.03)
	L24950	Dec. 2018	2.66 (0.20)	
	L22072	Oct. 2018	2.68 (0.23)	
Botox^®^ 100 U	C4321C3	Sep. 2019	0.89 (0.12)	0.90 (0.03)
	C4289C3	Sep. 2019	0.89 (0.10)	
	C4270C3	Aug. 2019	0.94 (0.09)	
Xeomin^®^ 100 U	694458	Jul. 2019	0.41 (0.07)	0.40 (0.01)
	696232	Sep. 2019	0.40 (0.05)	
	694788	Sep. 2019	0.40 (0.05)	

BoNT-A, Botulinum neurotoxin type-A; ELISA, enzyme-linked immunosorbent assay; SD, standard deviation; U, units.

**Table 2:** Quantity of 150 kDa BoNT-A (pg) per manufacturer-assigned unit in Dysport^®^, Botox^®^, and Xeomin^®^.

Product	Batch	Expiry Date	Quantity of BoNT-A per Product Unit (pg/unit)	
			Calculated for Batch	Product, Mean (SD)
Dysport^®^ 500 U	M00405	Dec. 2018	5.45	5.38 (0.07)
	L24950	Dec. 2018	5.32	
	L22072	Oct. 2018	5.36	
Botox^®^ 100 U	C4321C3	Sep. 2019	8.86	9.04 (0.30)
	C4289C3	Sep. 2019	8.88	
	C4270C3	Aug. 2019	9.38	
Xeomin^®^ 100 U	694458	Jul. 2019	4.09	4.03 (0.06)
	696232	Sep. 2019	4.01	
	694788	Sep. 2019	3.97	

BoNT-A, botulinum neurotoxin type-A; SD, standard deviation; U, units.

**Table 3:** Total quantity of active 150 kDa BoNT-A in maximum recommended doses of BoNT-A products.

Indication	Product	A—Total Recommended Dosage ^a^, Product Units	B—Amount of Neurotoxin Per Product Unit, pg	C—Total Amount of Active BoNT-A (ng) Injected at the Recommended Dose, C = A × B
AUL	Dysport^®^	1000	5.38	5.38
	Botox^®^	400	9.04	3.62
	Xeomin^®^	400	4.03	1.61
ALL	Dysport^®^	1500	5.38	8.07
	Botox^®^	400	9.04	3.62
	Xeomin^®^		4.03	
GL	Dysport^®^	50	5.38	0.27
	Botox^®^ *	20	9.04	0.18
	Xeomin^®^	20	4.03	0.08

* Botox Cosmetic; ^a^ according to prescribing information [13,14,15,31]. ALL, adult lower limb; AUL, adult upper limb; BoNT-A, botulinum neurotoxin type-A; GL, glabellar lines.

**Table 4:** Quantity (ng) of 150 kDa BoNT-A in Dysport^®^, Botox^®^, and Xeomin^®^, analysed by ELISA using BoLISA^®^ antibodies, compared to an equivalent quantity using the EndoPep method^®^.

Product	Vial	Quantity of BoNT-A (ng/Vial) ELISA		Quantity of BoNT-A (ng/Vial) EndoPep	
		Per Vial	Product, Mean (SD)	Per Vial	Product, Mean (SD)
Dysport^®^ 300 U	1	1.87	1.81 (0.12)	1.45	1.42 (0.05)
	2	1.88		1.45	
	3	1.67		1.37	
Botox^®^ 100 U	1	0.97	0.89 (0.10)	0.85	0.96 (0.10)
	2	0.92		0.98	
	3	0.78		1.05	
Xeomin^®^ 100 U	1	0.46	0.44 (0.02)	0.39	0.35 (0.04)
	2	0.44		0.33	
	3	0.41		0.32	

BoNT-A, botulinum neurotoxin type-A; ELISA, enzyme-linked immunosorbent assay; SD, standard deviation; U, units.

**Table 5:** Relative light chain activity per ng of Dysport^®^, Botox^®^, and Xeomin^®^ analysed using the EndoPep method.

Product	Comparator	Difference in LC Activity, Mean (SE)	Z Score	*p* value	*p* value Adjusted	Significance
Dysport^®^	Botox^®^	0.293 (0.285)	1.0304	0.3028	0.6611	NS
Dysport^®^	Xeomin^®^	0.000 (0.176)	0.0014	0.9989	1.0000	NS
Xeomin^®^	Botox^®^	0.293 (0.237)	1.2375	0.2159	0.5179	NS

LC, light chain; NS, not significant; SE, standard error.

**Table 6:** Excipients of the various products and the corresponding reconstitution buffer for each product to ensure that all products are in the same matrix during the EndoPep assay.

Product	Serum Albumin (mg/mL)	Sucrose (mg/mL)	Lactose (mg/mL)	NaCl (mg/mL)
Xeomin^®^ product	5	23.5	0	0
Xeomin^®^ reconstitution buffer	0	0	12.5	4.5
Botox product	2.5	0	0	4.5
Botox^®^ reconstitution buffer	2.5	23.5	12.5	0
Dysport^®^ product	0.62	0	12.5	0
Dysport^®^ reconstitution buffer	4.38	23.5	0	4.5
r-BoNT-A reconstitution buffer	5	23.5	12.5	4.5

NaCl, sodium chloride; r-BoNT-A, recombinant botulinum neurotoxin type A.
