# Correction: Field, M. et al. AbobotulinumtoxinA (Dysport^®^), OnabotulinumtoxinA (Botox^®^), and IncobotulinumtoxinA (Xeomin^®^) Neurotoxin Content and Potential Implications for Duration of Response in Patients

**DOI:** 10.3390/toxins11020115

**Published:** 2019-02-13

**Authors:** Malgorzata Field, Andrew Splevins, Philippe Picaut, Marcel van der Schans, Jan Langenberg, Daan Noort, Daniel Snyder, Keith Foster

**Affiliations:** 1 Ipsen Bioinnovation, Abingdon OX14 4RY, UK; g.field@oxb.com (M.F.); andrew.splevins@ipsen.com (A.S.); 2 Ipsen Pharma, Cambridge, MA 02142, USA; philippe.picaut@ipsen.com; 3 TNO—CBRN Protection, 2288GJ Rijswijk, The Netherlands; marcel.vanderschans@tno.nl (M.v.d.S.); jan.langenberg@tno.nl (J.L.); daan.noort@tno.nl (D.N.); 4 Ipsen Biopharmaceuticals, Basking Ridge, NJ 07920, USA; dsnyder@revance.com

The authors wish to make the following corrections to their paper [1].

Daniel Snyder should be listed as the author of their paper and the fourth affiliation “Ipsen Biopharmaceuticals, Basking Ridge, NJ 07920, USA; dsnyder@revance.com” should be added.

In the first affiliation, the original email address of Malgorzata Field is gosia.olszowka@gmail.com. This correct email address should be replaced by g.field@oxb.com

The “Author Contribution” section should be replaced by “Conceptualisation, M.F., A.S., P.P., D.S. and K.F.; Formal analysis, M.F., M.v.d.S., J.L. and D.N.; Investigation, M.F. and M.v.d.S.; Methodology, M.F., A.S., P.P., K.F. and M.v.d.S.; Validation, M.F., M.v.d.S., J.L. and D.N.; Visualisation, M.F., M.v.d.S., J.L. and D.N.; Writing—review & editing, M.F., A.S., P.P., M.v.d.S., J.L., D.N. and K.F. M.v.d.S., J.L. and D.N. were only involved in the EndoPep analyses.”

The “Conflicts of Interest” section should be replaced by “A.S., K.F. and P.P. are employed by Ipsen Pharma. D.S. and M.F. are ex-employees of Ipsen Pharma. M.v.d.S., J.L. and D.N. are employed by TNO.”

The manuscript will be updated and the original will remain online on the article webpage. We apologize for any inconvenience caused to our readers.
